# Enhanced production of pectinase by *Aspergillusterreus* NCFT 4269.10 using banana peels as substrate

**DOI:** 10.1007/s13205-015-0353-y

**Published:** 2016-01-23

**Authors:** Bijay Kumar Sethi, Prativa Kumari Nanda, Santilata Sahoo

**Affiliations:** 1 Microbiology Research Laboratory, P. G. Department of Botany, Utkal University, Vani Vihar, Bhubaneswar, Odisha 751004 India; 2 Department of Botany, Saila Bala Women’s College, Cuttack, Odisha 753001 India; 3 MITS School of Biotechnology, 2 (P), Infocity, Patia, Chandaka Industrial Estate, Bhubaneswar, Odisha 751024 India

**Keywords:** *Aspergillus terreus*, Liquid static surface fermentation, OVAT, Solid state fermentation

## Abstract

*Aspergillus terreus* NCFT4269.10 was implemented in solid-state (SSF) and liquid static surface fermentation (LSSF) for biosynthesis of pectinase. Amongst various substrates, like, mustard oil cake, neem oil cake, groundnut oil cake, black gram peels, green gram peels, chickling vetch peels/grass pea peels wheat bran, pearl millet residues, finger millet waste, broken rice, banana peels (BP), apple pomace (AP) and orange peels, banana peel (*Musa paradisiaca* L.; Family: Musaceae) was most suitable for pectinase biosynthesis (LSSF: 400 ± 21.45 Uml^−1^; SSF: 6500 ± 1116.21 Ug^−1^). Optimization of process parameters using one-variable-at-a-time method revealed that an initial medium pH of 5.0 at 30 °C and 96 h of incubation along with mannitol, urea, ammonium persulfate and isoleucine have positive influence on pectinase production. Further, K^+^ (1 mM), Riboflavin (10 mg 100 ml^−1^) and gibberellic acid (0.025 %, w/v) supported in enhanced pectinase production. Banana peels and AP at a ratio of 9:1, moisture content of 90 % with 2 % inoculum size were suitable combinations for production of pectinase. Similarly, 96 h of soaking time with 0.1 M phosphate buffer (pH 6.5) is essential for pectinase recovery. Purification to electrophoretic homogeneity revealed 1.42 fold purification with 8.08 % yield and a molecular weight of 24.6 kDa. Scaling up of various fermentation parameters and supplementing BP as the substrate for pectinase production with better recovery could make it promising for different industrial exploitation.

## Introduction

In the industrial arena, pectinase, the catch-all idiom that refers to mixtures of primarily three different enzymatic activities [pectin Esterase (PE), polygalacturonase (PG) and pectin/pectate lyase (PL/PAL)] is produced by a variety of bacteria (Kashyap et al. 2001) and fungi (Huang and Mahoney 1999). Huge portions of the fungal glycoside hydrolases belongs to GH family 28 are associated with the biodegradation of pectin backbone (Martens-Uzunova and Schaap 2009). The enzymes that hydrolyze pectic substances are broadly known as pectinolytic enzymes or pectinases, which include polygalacturonase, pectin esterase, pectin lyase, and pectate lyase on the basis of their mode of action (Dinu et al. 2007). Pectinolytic enzymes are categorized on the basis of their cleavage of the galacturonan portion of the pectin molecule. They can be distinguished between pectinesterases (PE, E.C 3.1.1.11), which modify pectin esters into low methoxyl pectins or pectic acid and pectin deploymerases, that split the glycosidic linkages between galacturonosyl (methyl ester) residues. Polygalacturonases split glycosidic linkage next to free carboxyl groups by hydrolysis, while pectin and pectate lyases split α-1, 4-glycosidic linkages by transelimination ensuing in galacturonide with a double bond between C_4_ and C_5_ at the non-reducing end and smite the α-1, 4-allied d-galacturonic acid remainders within the smooth provinces of pectin through a β, α-elimination mechanism. Both these endo types of PGs and PLs (E.C 3.2.1.15 and E.C 4.2.2.2, respectively) are recognized as the arbitrary splitters of pectin chain. Exo-polygalacturonases (exo-PGs, E.C 3.2.1.67) liberate monomers or dimmers from the non-reducing side of the chain, whereas exopectate lyases (exo-PALs, E.C 4.2.2.9) discharge unsaturated dimers from the reducing end. Pectinases comprise a consortium of enzymes indispensable for the hydrolysis of pectin, which consist of endo-polygalacturonase (EC 3.2.1.15), exo-polygalacturonase (EC 3.2.1.67), pectate lyase (EC 4.2.2.2), Exo-poly α-galactouronosidase (E.C.3.2.1.82), pectin lyase (EC 4.2.2.10), Exo-pectatelyase (E.C.4.2.2.9), Endopectin lyase (E.C.4.2.2.10) and pectin methyl esterase (EC 3.1.1.11). Depending on the pH optima, pectinases can also be categorized into acidic and alkaline types.

At contemporary, agricultural and food wastes are the major sources of pollution in developing countries which is being controlled by biological degradation using microorganisms for the biosynthesis of valuable compounds such as proteins, polysaccharides, oligosaccharides, vitamins, hormones, enzymes and others as raw materials for medical and industrial exploitations (El-Sheikh et al. 2009). Several microbes are capable of using these substances as carbon and energy sources for the biosynthesis of a vast array of pectinolytic enzymes in different environmental niches. But, the best-acknowledged microbial producers of pectinase are various species of *Aspergillus* and *A.* *niger* is the most admired one (Godfrey and West 1996). The impending fungal species exploited for the biosynthesis of pectinase are *A. foetidus* (Sebastian et al. 1996), *A. niger* (Taragano et al. 1997), *A. awamori* (Blandino et al. 2002), *Rhizopus, Trichoderma, Penicillium* and *Fusarium* spp. (Zeni et al. 2011).

Pectinases, explicitly polygalacturonases (PG) have gained significant worldwide applications in food and textile industries, in the biosynthesis of fruit juice (Singh et al. 2005), plant tissue maceration, wastewater treatment, degumming of plant webbers without any damage to the end products (Kapoor et al. 2001) and processing of vegetables and fruits and for clarification of juices and wines (Pereira et al. 2002). To reiterate, commercially significant pectinases (PG) have been employed in preparation of apple juice for higher juice yield, clarity, colloid concentration and polyphenolic contents (Oszmianski et al. 2009), coffee and tea fermentations, oil extraction (Hoondal et al. 2000), purification of viruses (Salazar and Jayasinghe 1999), and enhancement of chromaticity and stability of red wines (Revilla and Jose 2003). It has also been successfully supplemented in concurrence with amylase, lipases, cellulases, and hemicellulases to get rid of sizing agents from cotton in a secure and eco-friendly approach (Hoondal et al. 2000). Altogether, pectinases from microbial sources contribute almost 25 % of world wide food enzyme trading (Tari et al. 2007).

Solid-state fermentation has been advocated superior than submerged culture for the processing of agricultural and agro-based industrial wastes for generating higher enzyme yield and regulating the biosynthesis of particular composition of enzyme mixtures (Patil and Dayanand 2006). The water activity (*a*_w_), pH, temperature, incubation time, moisture content, inoculums size, presence of inhibitors or activators, carbon and nitrogen sources are critical factors that also influence pectinase biosynthesis in solid-state fermentation (Taragano et al. 1997). Further, the selection and optimization of substrate and culture condition is another most imperative approach to trim down industrial costs of enzyme biosynthesis.

Keeping in view, this present work aims at the bio-utilization of banana peels for biosynthesis of pectinase by *Aspergillus* *terreus* NCFT4269.10. In this study, various process parameters were evaluated, optimized and established for enhanced biosynthesis of biotechnologically significant pectinase by *A. terreus* NCFT 4269.10 based on the medium composition and cultural conditions in tune with the effective utilization of banana peels and biosynthesis of pectinase for future promises.

## Materials and methods

### Substrates and chemicals

Various agro-waste residues like mustard oil cake (MoC), neem oil cake (NoC), groundnut oil cake (GnoC), black gram peels (BGP), green gram peels (GGP), chickling vetch peels/grass pea peels (CVP) wheat bran (WB), pearl millet residues (PMR), finger millet waste (FMW), broken rice (BR), banana peels (BP), apple pomace (AP), and orange peels (OP) were purchased from the market of Bhubaneswar, Odisha, India. Substrates were dried in the hot air oven at 60 °C up to 48 h, ground to fine powder, sieved and kept in sterile containers until used. All chemicals used in this study were of analytical reagent (AR) grade and purchased from Sigma, Hi-Media Limited, SRL Pvt. Limited and Merck India Limited (Mumbai, India).

### Source of fungal inoculum

A pectinase producing seven-day-old potato dextrose agar (PDA) slant culture of *Aspergillus terreus* (NCFT 4269.10) (Sethi et al. 2013a) was suspended in 5.0 ml of sterile deionised water. Then, 1.0 ml of spore suspension was used as the inoculum for pre-fermentation culture and incubated at 30 ± 1 °C for a week to obtain about 5.0 × 10^8^ spores ml^−1^. Finally, an initial spore density of 1 × 10^7^ spores ml^−1^ was used as inoculum for fermentation (Sethi et al. 2013b).

### Selection of suitable substrate for fermentation

Liquid static surface fermentation (LSSF) and solid state fermentation (SSF) were carried out using pre-processed substrates (10 g) as the constituents of fermentation medium. In Erlenmeyer flask of 150 ml capacity, sterilized fermentation medium (50 ml) having either MoC, NoC, GnoC, BGP, GGP, CVP, WB, PMR, FMW, BR, BP, AP, and OP as substrates was inoculated with 1 × 10^7^ spores ml^−1^ from 7 days old pre-fermentation culture broth and incubated at 30 ± 1 °C under static condition (Sethi et al. 2013b). Similarly, Erlenmeyer flasks (250 ml) containing each sterilized 5 g (w/w) of substrate and 8 ml of minimal salt solution were inoculated aseptically with optimum number of spore inoculum and incubated at 30 ± 1 °C with intermittent observation. After 96 h, fermented media (both LSSF and SSF) were processed for recovery of crude pectinase. Further, LSSF and SSF were carried out by taking the selected substrate for extracellular pectinase biosynthesis. The crude pectinase thus obtained was preserved at −20 °C for subsequent experiments. Dry weight of the biomass was determined after drying at 80 °C in hot air oven (Wadegati Instruments Ltd., Mumbai) up to 24 h.

### Optimization of process parameters

Various environmental, nutritional and other fermentation parameters were established for enhanced production of pectinase using banana peel as the fermentation medium by SSF and LSSF. The process parameters optimized were pH (3–10), temperature (24–45 °C @ 3 °C interval), incubation time (24–168 h), additional carbon sources (1.0 %, w/w), nitrogen sources (organic and inorganic, 1.0 %, w/w), amino acids (1.0 mM), metal ions (1.0 mM), antioxidant vitamins (10–50 mg 100 ml^−1^), growth hormones (0.0025 mgg^−1^, w/w), combined agro-wastes, inoculum size (2–10 %) and initial moisture content (20–100 %). The effect of soaking time on fermented products (1–96 h), repeated extraction and various extractants were also standardized for the ease and economic down-streaming of pectinase. The approach adopted was to scale up one-variable-at-a-time (OVAT), independent of the others and consequently established conditions were adopted in rest of the experiments.

### Purification of pectinase

The crude culture filtrate (~500 ml) was precipitated by gradual addition of 40–80 % ammonium sulfate [(NH_4_)_2_S0_4_] with constant stirring by a magnetic stirrer at 4 °C up to 24 h. Each precipitate was separated from the supernatant by centrifugation at 10,000×*g* for 15 min at 4 °C. After centrifugation, the supernatant was decanted and the solid precipitate was dissolved in phosphate buffer (pH 6.5) at a ratio of 0.1 g ml^−1^ so as to obtain 10-times more concentrated enzyme solution (Jana et al. 2013). Thereafter, ammonium sulfate precipitated enzyme solution was dialyzed for 24 h at 4 °C with incessant stirring against a large volume (1 l capacity) of phosphate buffer (pH 6.5) for the complete removal of lower molecular weight metabolites and ammonium sulfate from the dialysate. To improve solute exchange, the dialysis buffer was replaced after every 2 h of incubation so as to ascertain a new concentration gradient. Then, the dialyzed enzyme was loaded into the Sephadex G-100 column (2.5 cm × 70 cm) and eluted with 50 mM phosphate buffer (pH 6.5) with the flow rate of 1 ml min^−1^. Fractions of 2 ml each were subsequently collected for estimation of protein (Lowry et al. 1951). The fractions showing maximum absorption at 750 nm were collected and evaluated for its enzyme activity. The enzyme positive fractions with higher enzyme activity were combined together, lyophilized and stored at −20 °C for further characterization.

### Analytical methods

#### Total protein determination

Crude and purified protein content was estimated as per Lowry et al. (1951) taking bovine serum albumin as the standard and was expressed as µg of protein present per ml of extract obtained after fermentation and purification.

#### Pectinase assay

The Standard protocol of Sigma Quality Control Department (Khairnar et al. 2009) was used for the enzyme assay. 0.5 % pectin solution (4.90 ml), 50 mM iodine with 200 mM potassium iodide (5.0 ml), 1 M sodium carbonate (1.0 ml), 2 M sulfuric acid (2.0 ml), 1 % pectin indicator (1–2 drops) and diluted pectinase solution (100 µl) were mixed thoroughly by swirling. The mixture (test and blank) was titrated with 100 mM sodium thiosulfate until it was turned to light yellow. To that, 1 drop of pectin indicator (starch) was added and continuously titrated until solution becomes colorless. One unit (U) of pectinase activity was defined as the amount of enzyme that releases 1 µg of product in 1 min under the assay conditions. The calculation for pectinase activity is presented below.

$${\text{Units}}/{\text{ml}}\,{\text{enzyme}} = \left[ {\left( 1 \right) \, \left( {100} \right)\left( {{\text{ml}}\,{\text{of}}\,{\text{sodium}}\,{\text{thiosulfate}}\,{\text{for}}\,{\text{blank}} - {\text{ml}}\,{\text{of}}\,{\text{sodium}}\,{\text{thiosulfate}}\,{\text{for}}\,{\text{test}}} \right)\left( {\text{df}} \right)} \right]/\left( 5 \right) \, \left( {0.1} \right) \, \left( 2 \right)$$

where, 1 = one μmole galacturonic acid is oxidized by 1 microequivalent of I_2_, 100 = microequivalents of S_2_O_3_/ml of reagent E, df = dilution factor, 5 = time of reaction in minutes, 0.1 = volume (in mm) of enzyme used, 2 = microequivalents of S_2_O_3_ oxidized per microequivalent of I_2_ reduced.

#### SDS-PAGE and Zymographic analysis

The samples (crude and purified pectinase) were electrophoresed in 10 % SDS-PAGE for molecular weight determination (Laemmli 1970). For visualization of bands, gels were stained in a staining solution [0.1 % (w/v) Coomassie-Brilliant Blue R-250, 50 % (v/v) methanol, 7 % (v/v) glacial acetic acid and 43 ml milli Q distilled water] for 90 min at room temperature followed by destaining with 30 % (v/v) methanol, 7 % (v/v) acetic acid and 63 ml milli Q distilled water until the background was clear. The relative positions of bands were analyzed using Bio-Rad Gel documentation system. Different molecular weight protein markers ranging from 7–175 kDa (Bangalore Genei Ltd.) were used for SDS-PAGE.

For native PAGE, enzyme sample was loaded with loading dye in a 10 % non-denaturing gel and run for 8 h in a discontinuous buffer system using 50 mM Tris-0.1 M glycine running buffer (pH 8.8) at 4 °C and 20 mA current. The gel was incubated for 16 h at 37 °C in 1 % soluble pectin prepared by 50 mM citrate–phosphate buffer (pH 5.0) followed by staining with 1 % congo red solution for 15 min. Stained gel was washed repeatedly with 1 M NaCl until the bands become clear (Garcia-Garrido et al. 2000). The gel image was taken and analyzed by Bio-Rad Gel doc system.

#### Fermentation kinetics study

For fermentation using agro-wastes by *A. terreus*, the logistic Eq. (1) and Luedeking-Piret model (Eq. 2) were employed for microbial growth and enzyme biosynthesis, respectively. It is as follows.

1 $${\text{d}}x/{\text{d}}t = \mu_{\hbox{max} } \left\{ {1 - \left( {x/x_{\hbox{max} } } \right)} \right\}x$$

2 $${\text{d}}P/{\text{d}}t = \alpha \mu x - \beta x$$

where dx/dt: biomass accumulation in the culture medium (g l^−1^ h^−1^); d*P*/d*t*: enzyme accumulation in the culture medium (U ml^−1^ h^−1^); ***x***: biomass (mg ml^−1^) at time (*t*);* µ*: specific growth rate (h^−1^);* µ*_max_: highest specific growth rate observed during batch culture (mgl^−1^ h^−1^);* x*_max_: maximum attainable biomass (mg ml^−1^);* α*: growth associated coefficient of enzyme production (U g^−1^);* β*: growth-independent coefficient of enzyme production (U g^−1^ h^−1^).

### Reproducibility of results

All experiments were carried out in triplicates (*n* = 3) and repeated three times. The samples collected from each replicate were analyzed for biosynthesis of the enzyme including its activity and optimization (scale up) of culture conditions. Each value is an average of three parallel replicates. The ± and error bars indicate standard deviation among the replicates. For each individual experiment, one way ANOVA was calculated using SPSS 16.0 workbook software. Least significant differences were also calculated using Duncan’s new multiple range tests.

## Results and discussion

### Selection of suitable substrate

The increasing energy demands have focused worldwide attention on the utilization of renewable resources, particularly agricultural and forest residues, the major components of which are cellulose, starch, lignin, xylan, and pectin. These materials have attracted considerable attention as an alternative feedstock and energy source, since they are abundantly available in developing countries like India. Several microbes are capable of using these substances as carbon and energy sources by producing a vast array of enzymes in different environmental niches. Although, a large number of organisms biosynthesizing pectinases have been documented, but, assortment of industrially pertinent microbes remains a tiresome job especially, when physiologically impending strains are to be isolated to attain utmost yield (Pandey et al. 1999). The most difficult, labor intensive task in designing the production process entirely relies on the nature of microorganism, fermentation system and the substrate chosen. Keeping in view, a preliminary screening was performed using commercial citrus pectin for the selection of a suitable pectinolytic fungus (Sethi et al. 2013a). A native fungal isolate, *Aspergillus* *terrues* NCFT 4269.10 was found to be superior in pectinase production as compared to the previously reported results (Patil et al. 2012; Maller et al. 2013). Therefore, this fungal species was used for the subsequent experiments. Nevertheless, commercial pectin as medium component may be too costly to accommodate in any fermentation process. Therefore, for selection of suitable and economic substrate for pectinase biosynthesis using fermentation, various low cost substrates like MoC, NoC, GnoC, BGP, GGP, CVP, WB, BR, PM, FM, BP, AP, and OP were evaluated individually as principal media components. Among the various substrates, BP was found suitable for noticeable production of pectinase (550 ± 70.71 Uml^−1^; 6500 ± 1116.21 Ugds^−1^) (Table 1). Most of the substrates could not support the remarkable biosynthesis of pectinase except AP and OP (Table 1). This can be attributed to the higher pectin content (10–21 %) of banana peels as compared to the different agro-wastes taken for the study. Further, banana peel is also prosperous in proteins, essential amino acids, dietary fibre, polyunsaturated fatty acids, iron, and potassium (Juarez-Garcia et al. 2006). The peels are rich in large quantities of dopamine, an antioxidants (80–560 mg per 100 g in peel) containing 9.14 % of N (González-Montelongo et al. 2010) which is the cause of enhanced production of pectinase. SSF system has been explored than SmF for high volume-high-valued pectinase production using a variety of solid residues, many of which are of agro-industrial residues in origin. They include apple peels (*Malus domestica*), orange peels (*Citrus sinensis*), lemon peels (*Citrus latifolia*), passion fruit peels (*Passiflora edulis*), commercial mate herb (*Illex paraguariensis*), rice straw (*Oryza sativa*), sugar cane bagasse (*Saccharum officinarum*), corn cob (*Zea mays*), wheat bran (*Triticum aestivum*), and soya bran (*Glycine max*) (Maller et al. 2011). Likewise, wheat bran (Abbasi and Mortazavipur 2011) and cocoa beans (Akintobi et al. 2012) were also utilized for biosynthesis of pectinase. The substrate specificity was also reported for polygalacturonases from *Mucor circinelloides* ITCC 6025 (Thakur et al. 2010) and *Aspergillus giganteus* (Pedrolli and Carmona 2010). Ramadas et al. (1996) have reported three fold higher enzymatic activity in solid-state fermentation compared to static culture fermentation using *A*. *niger* that supports the present findings. In the same vein, this present study on agricultural residues has also shown that SSF is significantly better as a cultivation regimen as compared to SmF for production of fungal pectinase on banana peels.

**Table 1:** Pectinase biosynthesis by *Aspergillus terreus* NCFT4269.10 in liquid static surface fermentation and solid state fermentation systems using various agricultural wastes as the substrates

Fermentation medium	Cultivation regimen	Pectinase activity	Total protein	Biomass (g 50 ml^−1^)
MoC	LSSF	100 ± 11.11	478.33 ± 23.75	0.203 ± 0.013
	SSF	0	21.75 ± 4.42	–
NoC	LSSF	0	1723 ± 211.84	0.094 ± 0.007
	SSF	0	3.13 ± 0.77	–
GnoC	LSSF	0	1759.99 ± 25.76	0.243 ± 0.002
	SSF	0	2593.33 ± 42.78	–
BGP	LSSF	0	2446.64 ± 39.61	0.085 ± 0.002
	SSF	0	1106.64 ± 11.27	–
GGP	LSSF	0	4229.99 ± 56.24	0.157 ± 0.004
	SSF	0	3889.98 ± 47.34	–
CVP	LSSF	0	2266.98 ± 28.44	0.2108 ± 0.006
	SSF	0	2066.33 ± 21.11	–
WB	LSSF	0	458.98 ± 11.72	0.080 ± 0.001
	SSF	0	229.99 ± 27.41	–
PM	LSSF	0	246.19 ± 3.76	0.1412 ± 0.212
	SSF	0	288.98 ± 5.66	–
FM	LSSF	500 ± 47.66	446.64 ± 11.91	0.032 ± 0.004
	SSF	4000 ± 256.91	106.64 ± 7.37	–
BR	LSSF	0	759.99 ± 5.073	0.1981 ± 0.0812
	SSF	0	593.33 ± 3.121	–
BP	LSSF	550 ± 70.71	1234.99 ± 57.97	0.017 ± 0.044
	SSF	6500 ± 1116.21	1478.33 ± 76.44	–
AP	LSSF	400 ± 21.45	4488.98 ± 112.56	0.2117 ± 0.0217
	SSF	4000 ± 276.82	4467.33 ± 125.42	–
OP	LSSF	250 ± 21.77	1759.99 ± 89.65	0.031 ± 0.065
	SSF	4000 ± 276.93	2459.98 ± 124.76	–
Control^a^	LSSF	100 ± 11.79	1234.99 ± 48.22	0.0804 ± 0.001

The liquid static surface fermentation (LSSF) and solid state fermentation (SSF) experiments were performed for 96 h at 30 °C. The data represent mean ± SD of replicates (*n* = 3). {Uml-1, µg/ml and µmol/ml are used for LSSF; Ugds-1, mg/gds and µmol/gds are used for SSF}

[Weight of biomass (W3) = weight of biomass and filter paper (W1)–only weight of filter paper (W2)]

^a^Control refers to the basal medium with 1 % (w/v) commercial pectin as the inducer of pectinase

### Scale up of process parameters for enhanced biosynthesis of pectinase

#### Effect of initial medium pH

**Fig. 1:**
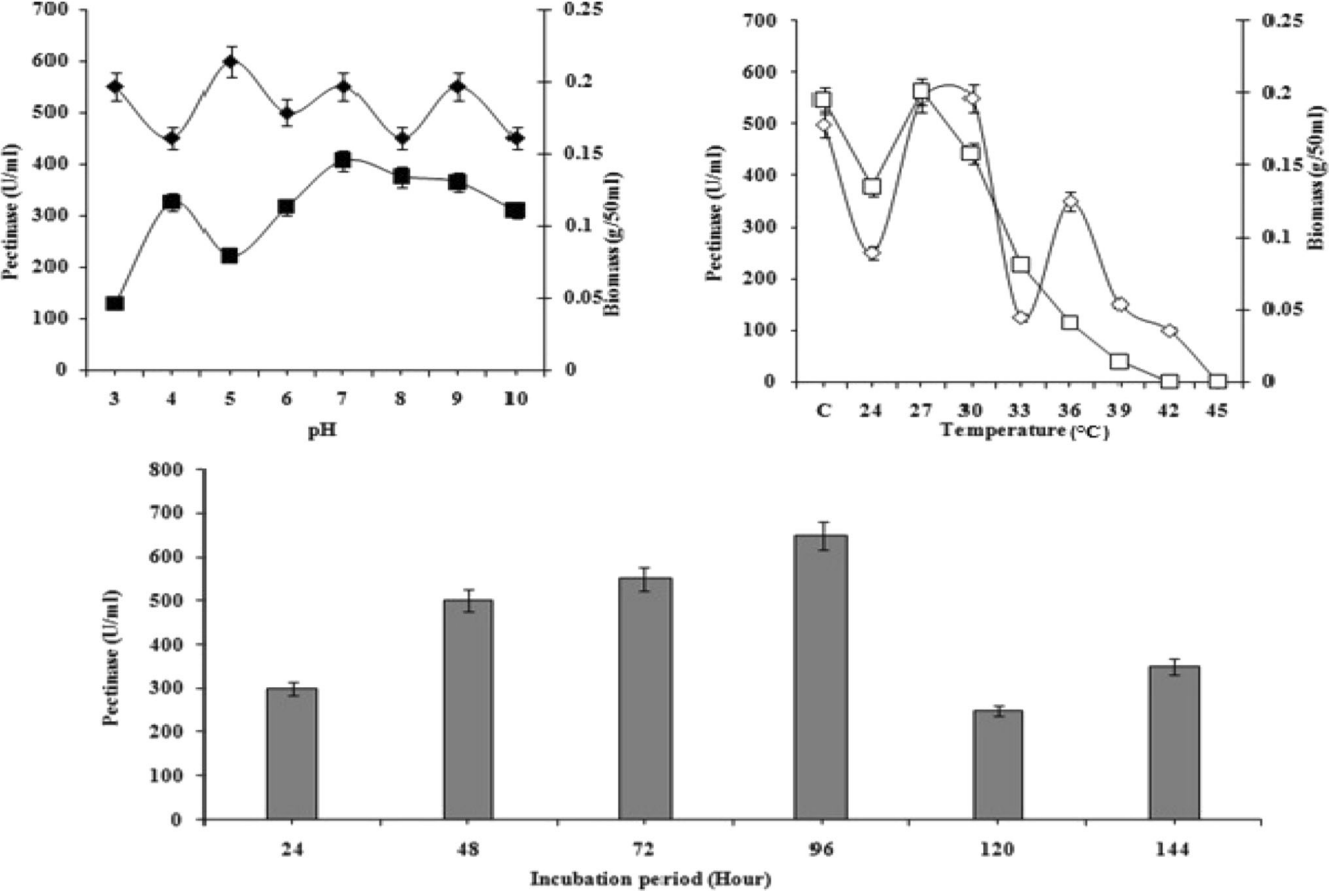
Effect of pH (3.0–10.0), incubation time (24–144 h) and temperature (24–45 °C @ 3 °C interval) on pectinase production and mycelia growth in liquid static surface culture (LSSF) by *Aspergillus terreus* NCFT 4269.10 (for pH, biomass of *A. terreus*: *filled square*; pectinase: *filled diamond*; for temperature, biomass of *A. terreus*: *open square*; pectinase: *open diamond*)

For enhanced production of pectinase by *A. terreus* NCFT 4692.10, pH 5.0 was found to be suitable (Fig. 1) which is supported by the findings of Mrudula and Anitharaj (2011) and Adeleke et al. (2012). But Patil et al. (2012) reported that pH 6.0 was optimum for production of pectinase by *Paecilomyces* *variotii* NFCCI 1769. As this enzyme was synthesized maximum at pH 5.0, hence can be an acidic pectinase (Kashyap et al. 2001) and it is polygalacturonase/pectin depolymerase/pectinase (E.C. 3.2.1.15). A decrease in final pH of the medium was observed in most of the cases. The pH of the medium also regulates the growth of the culture or exerts impact upon catalytic activity of the enzyme. According to Zeni et al. (2011), the acidification or alkalization of culture medium reflects the substrate consumption. Due to this, relation between the synthesis of polygalacturonases and the consumption of nitrogen compounds, the change of pH can be used to conclude with important information about the enzyme production with commencement and retardation of its synthesis.

#### Effect of temperature

Keeping the pH of the fermentation medium constant (pH 5.0), the LSSF was carried out at varying range of temperature (24–45 °C; an interval of 3 °C) for 96 h. Biosynthesis of pectinase and mycelial growth were utmost at 30 °C (Fig. 1). The enzyme, pectinase biosynthesis by *A*. *terreus* is in accordance with the report of Maciel et al. (2011). Similarly, for pectinase biosynthesis, Patil et al. (2012), Adeleke et al. (2012) and Maller et al. (2013) reported that 40 °C temperature was suitable which disagrees with the present findings. Mrudula and Anitharaj (2011) have also documented an optimum biosynthesis of pectinase using orange peels by *Aspergillus niger* at 50 °C. Nevertheless, the assessment of enzyme activities biosynthesized by numerous microorganisms is complicated, while discrete culture parameters and enzyme activity determinations have been employed (Maciel et al. 2011).

#### Effect of incubation time on biosynthesis of pectinase

At constant pH and temperature, the liquid static surface fermentation was performed at varying incubation period and concluded that maximum production was attained at 96 h of incubation though the biosynthesis started at 24 h. Meanwhile, the generation of biomass initiated within 24 h and gradually increased up to 144 h without showing any decline in growth profile. Further, the biosynthesis of enzyme was not strongly correlated with biomass production (Fig. 1). Maller et al. (2011) have also reported that production of maximum pectinase by *A*. *niveus* was recorded at 96 h of incubation which is in accordance with the current findings. Similarly, Mrudula and Anitharaj (2011) reported an optimum production of pectinase using orange peels as the agro-wastes by *Aspergillus niger* at 50 °C, pH 5 and 96 h of incubation. Patil et al. (2012) reported that maximum exo-polygalacturonase activity was obtained on the third day of incubation at 40 °C which differs from the present findings. Adeleke et al. (2012) concluded that *Penicillium atrovenetum*, *Aspergillus flavus* and *A. oryzae* produced polygalacturonase optimally on the 5th day while endoglucanase was produced optimally on the 7th day. The present finding was somehow different from the above reports and this might be due to the nature of substrate that induces the production of the enzyme at early late phase.

#### Effect of carbon sources on production of pectinase

**Fig. 2:**
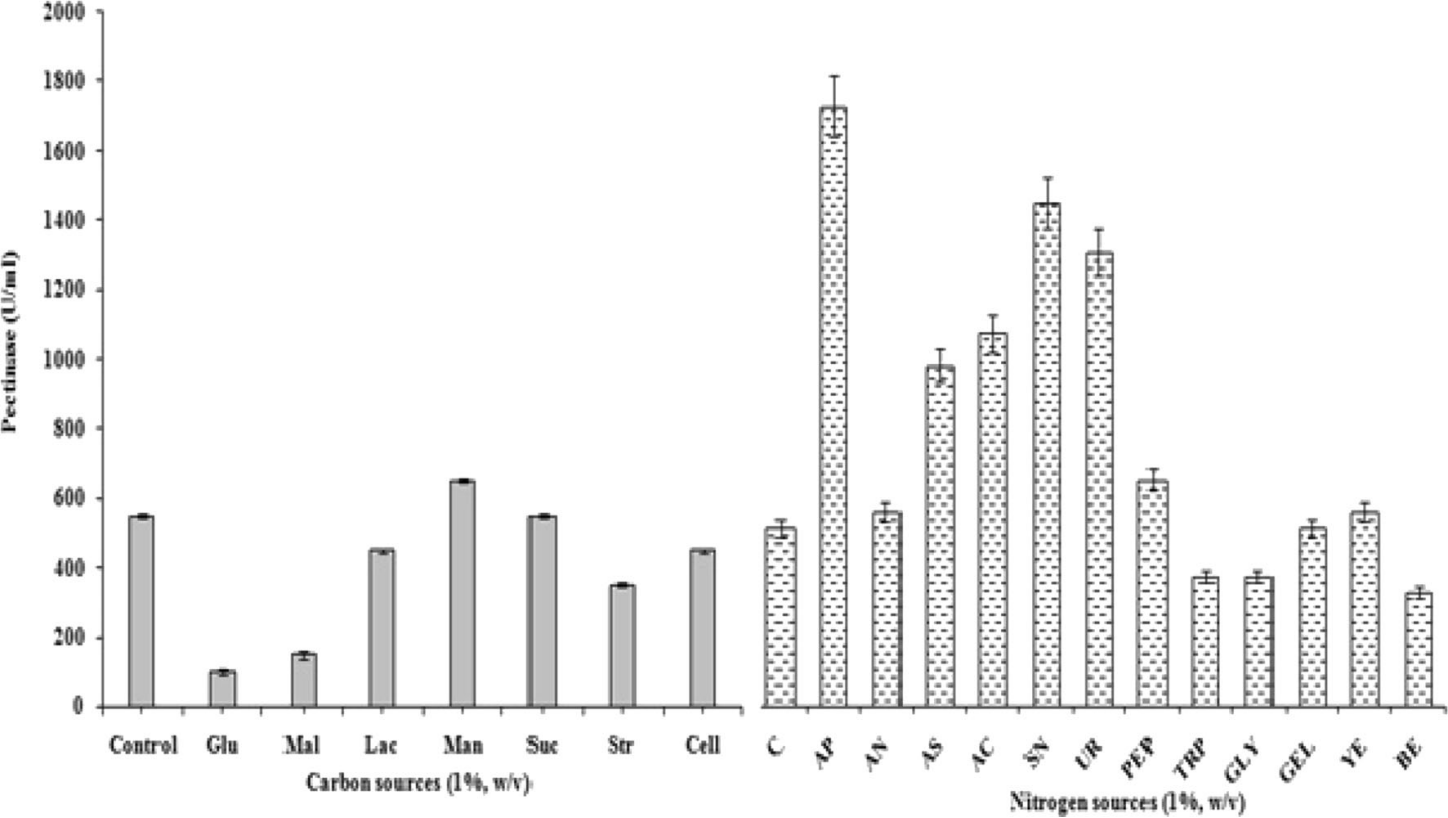
Effect of various carbon and nitrogen sources on the mycelia growth and pectinase production by *Aspergillus terreus*, where, *Glu* glucose, *Mal* maltose, *Lac* lactose, *Man* mannitol, *Suc* sucrose, *Str* starch, *Cell* cellulose, *AP* ammonium persulfate, *AN* ammonium nitrate, *AS* ammonium sulfate, *AC* ammonium chloride, *SN* sodium nitrate, *UR* urea, *Pep* peptone, *Trp* tryptone, *Gly* glycine, *Gel* gelatin, *YE* yeast extract and *BE* beef extract

Pectinase was produced at constant pH and temperature for 96 h with supplementation of additional carbon sources. It was concluded that pectinase production was enhanced when mannitol was served as the additional source of carbon. Rest of the carbon sources was not appropriate for biosynthesis of pectinase. Almost half of the activity was reduced as compared to the control when starch was supplemented with the banana peels as the nutrient supplement (Fig. 2). Starch as a substrate could not be able to support the secretion of pectinase is also reported by Arotupin (2007). Mrudula and Anitharaj (2011) reported an optimum production of pectinase on orange peels by *Aspergillus niger* when supplemented with sucrose. Similarly, Abbasi and Mortazavipur (2011) reported glucose and pectin supplementation along with wheat flour enhanced the Exo-PGase activity. This ability of *A. terreus* indicates that polygalacturonase production is not only inducible but constitutive.

#### Effect of nitrogen sources on production of pectinase

Out of the five organic nitrogen sources, peptone was found to be the best organic nitrogen source followed by yeast extract for exuberant biosynthesis of pectinase. Among all inorganic nitrogen sources evaluated, ammonium persulfate was most suitable for production of pectinase followed by sodium nitrate and urea. About three-fold increase in pectinase activity was attained with ammonium persulfate. All inorganic nitrogen sources have positive influence on pectinase biosynthesis indicating that the enzyme synthesis can be enhanced by supplementing cost-effective inorganic nitrogen sources (Fig. 2). In a study by Adeleke et al. (2012), highest production of polygalacturonase and endoglucanase by *Penicillium atrovenetum* was observed at pH 5, 40 °C and at 0.2 % ammonium persulfate which supports the present finding with maximum synthesis of pectinase (Fig. 2). Similar finding was also obtained by Akhter et al. (2011). Among the organic nitrogen substrates utilized peptone supported pectinase yield and can be comparable with the results reported by Akhter et al. (2011). This is in consonance with Arotupin (2007) who reported that organic nitrogen sources maintain the growth of fungi more than inorganic nitrogen sources. During growth, the fungi probably hydrolyze the organic nitrogen releasing their mineral component and other growth factors present in them into constituents that can be easily incorporated into the cellular metabolism.

#### Effect of amino acids on production of pectinase

In the present study, seventeen amino acids were supplemented to the fermentation medium and found that isoleucine at a concentration of 1 mM enhanced three-fold biosynthesis of pectinase, whereas, maximum biomass was attained with proline (Table 2). Demir et al. (2011) suggested that l-cysteine and ascorbic acid significantly enhanced pectinase activity in *Geobacillus* *stearothermophilus* which differs from the present findings.

**Table 2:** Effect of amino acids, metal ions, vitamins and growth regulators on the mycelia growth and pectinase production by *Aspergillus terreus* NCFT 4269.10^a^

Different sources	Pectinase		
	Biomass (g/50 ml)	Activity (Uml^−1^)	pH^b^
*Amino acids (5* *mM/100* *ml)*			
Alanine	0.45 ± 0.001^bc^	1150 ± 71^ef^	4.3
Proline	0.78 ± 0.007^a^	450 ± 71^jk^	5.6
Valine	0.52 ± 0.006^b^	800 ± 14^1h^	4.6
Aspartic acid	0.41 ± 0.005^c^	700 ± 14^1i^	4.2
Methionine	0.67 ± 0.228^ab^	750 ± 71^ih^	4.8
Glutamate	0.35 ± 0.007^c^	350 ± 71^k^	3.9
l-lycine	0.39 ± 0.008^c^	950 ± 71^g^	4.4
Cysteine	0.39 ± 0.007^c^	850 ± 71^gh^	4.4
Histidine	0.42 ± 0.004^c^	650 ± 71^j^	4.2
Phenyl alanine	0.32 ± 0.007^c^	850 ± 71^gh^	4.7
Isoleucine	0.49 ± 0.031^bc^	1700 ± 141^b^	7.7
Threonine	0.32 ± 0.001^c^	1400 ± 141^d^	4.2
Tryptophan	0.46 ± 0.009^bc^	200 ± 0.00 ^l^	4.1
Agrinine	0.28 ± 0.007^d^	850 ± 71^gh^	4.9
Leucine	0.37 ± 0.000^bc^	250 ± 71^kl^	4.3
Glycine	0.35 ± 0.000^c^	1150 ± 71^ef^	4.7
Serine	ND	ND	ND
*Metal ions (1* *mM)*			
Zn^2+^	0.05 ± 0.001^h^	350 ± 71^kl^	4.9
K^+^	0.12 ± 0.051^e^	1650 ± 71^bc^	4.9
Ag^2+^	0.08 ± 0.002^f^	50 ± 71^m^	4.4
Fe^2+^	0.11 ± 0.012^e^	250 ± 71^l^	4.8
Mg^+^	0.15 ± 0.011^e^	200 ± 71^l^	4.7
Cu^2+^	0.13 ± 0.014^e^	450 ± 71^k^	4.2
Mn^+^	0.13 ± 0.011^e^	1250 ± 141^e^	4.4
Ca^+^	0.11 ± 0.000^e^	200 ± 71^l^	5.1
Hg^+^	0.05 ± 0.001^g^	750 ± 141^ih^	5.0
EDTA	0.12 ± 0.016^e^	400 ± 71^k^	4.7
*Vitamins (mg/100* *ml)*			
Vitamin C			
10	1.89 ± 0.00^b^	3650 ± 212^a^	6.9
20	1.95 ± 0.03^a^	3150 ± 71^e^	6.4
30	2.03 ± 0.07^a^	3250 ± 71^d^	6.1
40	1.89 ± 0.12^b^	3350 ± 212^cd^	6.4
50	1.99 ± 0.02^a^	3350 ± 212^cd^	6.5
Riboflavin			
10	1.98 ± 0.01^a^	3700 ± 141^a^	6.2
20	1.81 ± 0.08^cd^	3350 ± 212^c^	6.6
30	1.59 ± 0.13^e^	3250 ± 71^d^	6.6
40	1.52 ± 0.08^e^	3150 ± 71^e^	6.7
50	2.11 ± 0.02^a^	3450 ± 71^b^	6.2
Folic acid			
10	1.64 ± 0.05^de^	3400 ± 141^bc^	5.2
20	1.45 ± 0.03^ef^	3250 ± 71^ce^	5.1
30	1.54 ± 0.06^e^	3250 ± 71^cd^	5.4
40	1.48 ± 0.02^e^	3350 ± 212^c^	5.3
50	1.12 ± 0.02^g^	3250 ± 71^cd^	5.5
Vitamin E			
10	1.78 ± 0.12^d^	3400 ± 141^c^	6.3
20	1.89 ± 0.01^b^	3450 ± 71^bc^	6.2
30	1.37 ± 0.01^f^	3250 ± 71^d^	5.7
40	1.42 ± 0.01^ef^	3350 ± 71^bc^	6.3
50	1.04 ± 0.00^g^	3300 ± 141^c^	6.4
*Growth regulators (0.0025* *mg/g, w/w)*			
Gibberellic acid	1.08 ± 0.16^a^	1400 ± 100^a^	5.2
Kinetin	1.03 ± 0.13^a^	950 ± 50^bc^	4.7
6-Benzylaminopurine	0.93 ± 0.21^b^	700 ± 100^c^	4.9
2,4-Dichlorophenoxyacetic acid	0.47 ± 0.08^c^	200 ± 58^d^	5.3

The data represent mean ± SD of replicates (*n* = 3). Mean values within a column with different upper-script letters are significantly different at *p* ≤ 0.05

^a^The liquid static surface fermentation experiments were performed for 4 days at 30 °C for all the cases

^b^pH: initial pH was adjusted 7.0 required for the biosynthesis of pectinase

#### Effect of metal ions

In the present study, it was observed that K^+^ has positive influence on biosynthesis of pectinase in comparison to other metal ions. Nonetheless, Cu^2+^, Zn^2+^ and Ca^2+^ have supported in biomass production. Addition of K^+^ to the fermentation medium at pH 5.0, temperature 30 °C and incubation of 96 h exhibited about three-fold higher pectinase biosynthesis (Table 2). This is also authenticated that pectinase may be a metalloenzyme, which is activated in the presence of metals, especially, the K^+^. JagadeeshBabu and Viswanathan (2010) have reported that significant enhancement in the level of pectinase production by *A.* *foetidus* NCIM 505 was achieved with Cu^2+^ which was found to be deleterious for *A*. *terreus*.

#### Effect of vitamins on biosynthesis of pectinase

The influence of various vitamins on the production of biomass and pectinase using *A. terreus* was evaluated by supplementing with banana peel medium at LSSF and found that riboflavin at 10 mg/100 ml concentration exhibited ~sevenfolds pectinase activity (3700 ± 53.03 Uml^−1^) (Table 2). Maximum biomass was obtained with riboflavin when supplemented at 50 mg/100 ml concentration to the fermentation medium. Afifi et al. (2008) also reported that vitamin C had the most pronounced effect on growth, protein accumulation and pectinase production by *P. olsonii* which deviates from the present findings. Similarly, vitamin C and riboflavin stimulated the synthesis of pectinase. From the results obtained with *A. terreus,* it is suggested that supplementation of vitamins is not an absolute demand for the production of pectinase and growth. Similar observations were also reported with *Volvariella esculanta* (Jonathan et al. 2004) and *V. speciosa* (Fasidi and Akwakwa 1996), the edible mushrooms of Nigeria.

#### Effect of plant growth hormones on biosynthesis of pectinase

To determine the effect of growth hormones (BAP, 2–4 D, Kinetin and gibberellic acid) on production of the enzyme, fermentation medium was supplemented with various growth hormones at the concentration of 0.025 % (w/v) after sterilization. Except 2–4, D, all the rest three supported in secretion of pectinase as well as production of protein and biomass (Table 2). Negi and Banerjee (2010) also studied the effect of growth hormones on production of pectinase and protease and observed that indol acetic acid (IAA) and Indol-3-butyric acid (IBA) stimulated production of both the enzymes produced by *A. awamori*. The report of Negi and Banerjee (2010) indicates that napthelene acitic acid (NAA) and 2–4, D enhanced production of protease but on the other hand, biosynthesis of pectinase was reduced.

#### Effect of mixed substrates on enzyme production

Various agro-wastes were mixed at different proportions and evaluated for their suitability to enhance the production of pectinase using SSF. It was concluded that BP: AP @ 9:1; BP: OP @ 3:7 exhibited highest pectinase production (Table 3). Similarly, Adeleke et al. (2012) evaluated the potential of the fungi to biosynthesize pectinase and cellulase employing orange peels as substrates. Mixture of orange bagasse and wheat bran was the best substrate for the production of pectinase in solid state fermentation using a *Penicillium* sp.

**Table 3:** Effect of mixed agro-wastes on pectinase biosynthesis

Mixed substrates	Ratio	Pectinase activity (Ugds^−1^)
Banana peels : orange peels	1:9	8500 ± 707.1^bc^
	3:7	10,000 ± 0.00^a^
	5:5	8500 ± 707.1^c^
	7:3	7500 ± 707.1^d^
	9:1	9000 ± 0.00^bc^
Banana peels: apple pomace	1:9	4500 ± 707.1^g^
	3:7	4000 ± 0.00^g^
	5:5	8500 ± 707.1^c^
	7:3	9500 ± 707.1^b^
	9:1	10,500 ± 707.1^a^
Orange peels : apple pomace	1:9	7000 ± 1414.2^de^
	3:7	6500 ± 707.1^e^
	5:5	7000 ± 1414.2^de^
	7:3	9500 ± 707.1^bc^
	9:1	7000 ± 1414.2^d^
Control^a^	–	5500 ± 707.1^f^

^a^Control refers to only BP taken as solid state fermentation medium (10 % w/v). The solid state fermentation setups were performed as per the above combination for a period of 96 h at 30 °C. The data represent mean ± SD of replicates (*n* = 3). Mean values within a column with different upper-script letters are significantly different at *p* ≤ 0.05

#### Effect of inoculum size

To determine the effect of inoculum size on production of enzyme, 2–10 % (v/w) inoculum was used in SSF medium by spore counting with haemocytometer and spectrophotometric analysis and evaluated for the production of enzyme. With 2 % inoculum, the biosynthesis of pectinase was maximum. However, further increase in size of inoculum resulted in decreased enzyme synthesis, probably due to depletion of the nutrients in the fermented medium. Mrudula and Anitharaj (2011) reported an optimum production of pectinase on orange peels by *Aspergillus niger* using 2.5 ml inoculums size. Maciel et al. (2011) have also reported that 1 × 10^7^ sporesg^−1^ was best for maximum synthesis of pectinase which was also similar with the report obtained from *A*. *terreus*.

#### Effect of initial moisture content

**Fig. 3:**
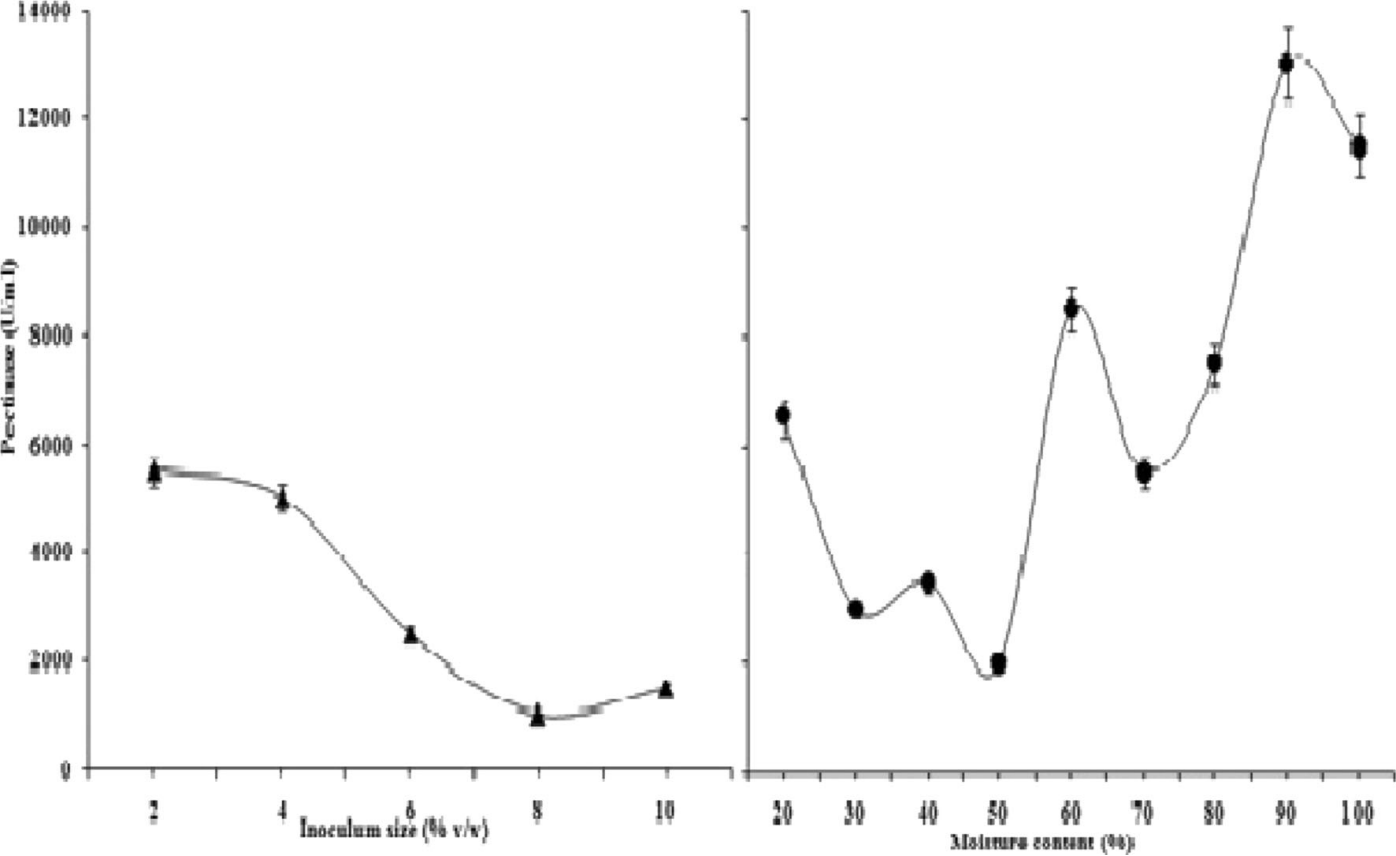
Effect of inoculum size and initial moisture content on biosynthesis of extracellular pectinase by *Aspergillus terreus* NCFT 4269.10 performed for 96 h at 30 °C using solid state fermentation

At 90 % moisture content optimum pectinase was produced (Fig. 3). But, Maller et al. (2011) reported 67 % humidity is suitable for biosynthesis of pectinase which is different in case of *A. terreus* (Fig. 3). Mrudula and Anitharaj (2011) reported an optimum production of pectinase using orange peels by *Aspergillus niger* with 1:2 % (v/w) moisture.

#### Down streaming of pectinase

After the successful SSF, extraction of pectinase was an obligatory step in achieving maximum recovery of this enzyme. Hence, the fermented matter was soaked with phosphate buffer (0.1 M) at varying interval of time to standardize an efficient soaking time for maximum release of enzyme. To extract the pectinase, fermented matter was soaked with phosphate buffer and 48 h of soaking was most suitable for maximum recovery of the enzyme. Repeated extraction with buffer facilitated the release of pectinase. However, at second time extraction with phosphate buffer, the release of enzyme was better than the first extraction. Among nine extractants, phosphate buffer (pH 6.5; 0.1 M) was most suitable in the release of enzyme. Other extractants were less supportive for efficient release of pectinase (Table 4).

**Table 4:** Effect of soaking time, repeated extraction and various extractants on pectinase recovery

Extraction	Pectinase activity		
	Ugds^−1^	Umg^−1^	Ug^−1^
*Soaking time (h)*			
0.5	2500 ± 710^f^	963 ± 270^h^	500 ± 140^g^
1	3500 ± 710^e^	1251 ± 190^g^	700 ± 140^f^
3	4500 ± 710^d^	1954 ± 300^f^	900 ± 140^e^
5	5500 ± 710^c^	2574 ± 220^e^	1100 ± 140^d^
7	7500 ± 710^b^	3736 ± 220^c^	1500 ± 140^b^
17	6500 ± 710^bc^	3592 ± 690^c^	1300 ± 140^c^
24	5500 ± 710^c^	2541 ± 260^e^	1100 ± 140^cd^
48	12,500 ± 710^a^	5297 ± 420^a^	2500 ± 140^a^
72	12,000 ± 0^a^	4727 ± 130^b^	2400 ± 140^a^
96	11,500 ± 710^a^	4652 ± 230^ab^	2500 ± 140^a^
*Repeated extraction*			
1	6500 ± 710^bc^	870.8 ± 270^i^	700 ± 140^f^
2	7500 ± 710^b^	3280 ± 410^d^	1500 ± 140^b^
3	5500 ± 710^c^	3726 ± 210^c^	1100 ± 140^d^
*Different extractants*			
PB (0.1 M)	5500 ± 710^c^	1992 ± 310^f^	1100 ± 140^d^
PB +Trit × 100 (0.1 %, w/v)	4500 ± 710^d^	2066 ± 280^ef^	900 ± 140^e^
PB +Trit × 100 (0.5 %, w/v)	3500 ± 710^e^	1187 ± 210^gh^	700 ± 140^f^
PB +Trit × 100 (1 %, w/v)	3000 ± 710^ef^	865.4 ± 310^i^	600 ± 140^fg^
PB + Urea (1 M)	2500 ± 710^f^	599.4 ± 340^j^	500 ± 140^g^
PB + Amm. Sulfate (1 M)	3500 ± 710^de^	1260 ± 220^g^	700 ± 140^f^
NaCl (0.5 %)	2500 ± 710^f^	1098 ± 180^h^	500 ± 140^g^
DW	3500 ± 710^d^	993 ± 230^h^	700 ± 140^f^
Czapek Dox	3000 ± 710^e^	886 ± 120^i^	600 ± 140^fg^

The solid state fermentation setups were performed for a period of 96 h at 30 °C. *ND* not determined. The data represent mean ± SD of replicates (*n* = 3). Mean values within a column with different upper-script letters are significantly different at *p* ≤ 0.05

#### Purification of pectinase

When gel filtration was employed for the pectic enzyme of *A. terreus*, only one absorption peak was obtained with 1.42 fold purification and 8.08 % yield with an increase in the specific activity from 445.35 to 634.73 Umg^−1^ (Table 5). Maller et al. (2013) purified pectinase from *Aspergillus* *niveus* using DEAE-cellulose followed by Biogel P-100 column and resulted in PG purification approximately 4.4-fold with 17.4 % recovery. While working with *Paecilomyces* *variotii* NFCCI 1769, Patil et al. (2012) reported that overall purification of pectinase was about 41.91 fold with a recovery of 26.90 %.

**Table 5:** Purification summary of isolated pectinase

Steps	Volume	Activity (Uml^−1^)	Total pectinase activity (U)	Total protein (mg)	Specific activity (Umg^−1^)	Purification fold	Yield (%)
Crude	422	550	232,100	521.165	445.35	1.0	100
Ammonium sulfate precipitation	24	950	22,800	44.64	510.75	1.15	9.82
Sephadex G-100	15	1250	18,750	29.54	634.73	1.42	8.08

#### Molecular weight determination by SDS-PAGE and Zymographic analysis

**Fig. 4:**
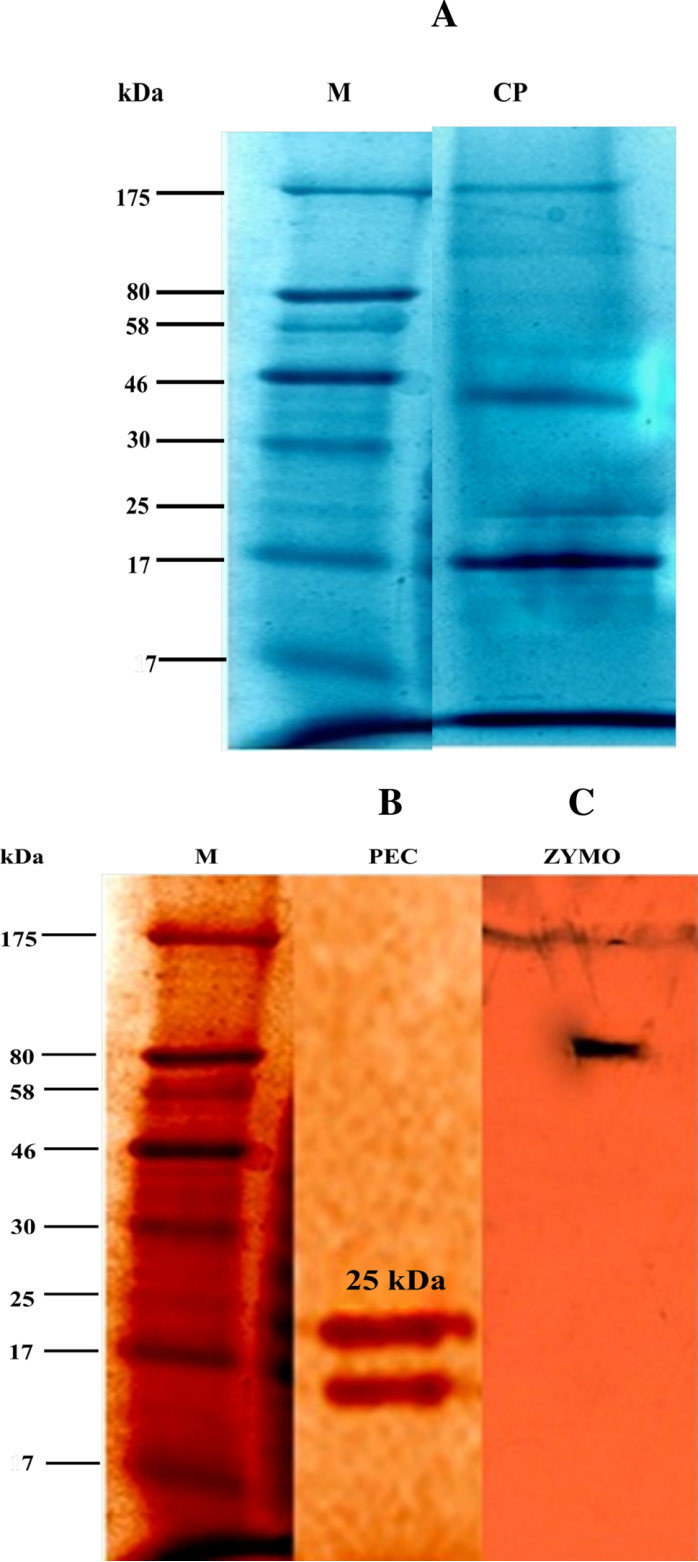
Electrophoretic analysis of pectinase **a** (*M* marker, *CP* crude pectinase), **b** (*M* marker, *PEC* purified pectinase), **c** (*ZA* zymographic analysis of pectinase)

The bands produced by the crude extracts of pectinase ranged from 7 to 175 kDa. But, the molecular weight of purified pectinase was found to be ~25 kDa (Fig. 4). Enzymatic activity was confirmed in native PAGE (zymographic analysis) that showed a unique degradation of sodium polypectate band. Reports also describe PG with high molecular mass as 60–70 kDa from *Aspergillus* *giganteus* (Pedrolli and Carmona 2010).

#### Fermentation kinetics study

Data obtained from above experiment was subjected to kinetics analysis for the calculations of *μ*_max_ (h^−1^) (specific growth rate), qp (unit product produced/g spores s/h), qs (g substrate consumed/g spores s/h), Yp/s (unit product produced/g substrate consumed), Yp/x (unit product produced/g spores s formed), Yx/s (g spores s/g substrate utilized), Qp (g spores s produced/l/h) and Qs (g substrate consumed/l/h). The kinetic evaluation results also revealed that the optimum fermentation period for extracellular biosynthesis of pectinase by *A. terreus* was 96 h with a constant growth rate. Nonetheless, growth and enzyme secretion were significantly affected by engineering of the fermentation media (supplementation of nutrients) and optimization of different parameter. The specific production rate and growth coefficient revealed hyperproducibility of extracellular pectinase (Table 6). Similar type of observation was also reported by Iftikhar et al. (2010) for production of lipase from *Rhizopus oligosporus* var. *microsporus*.

**Table 6:** Fermentation kinetics of biosynthesized pectinase

Optimum conditions	Y_e/s_ (Ug^−1^)	Y_x/s_ (gg^−1^)	*α* (Ug^−1^)	*β* (Ug^−1^ h^−1^)	q_c_ (mgg^−1^ h^−1^)	d*x/*d*t* (gL^−1^h^−1^)	d*P*/d*t* (Uml^−1^ h^−1^)	*x* (mg ml^−1^)	*µ*_max_ (mgl^−1^ h^−1^)
pH (5.0)	120	0.015	7594.93	1.25	3.29	0.016	6.25	1.58	16.46
Temperature (30 °C)	110	0.031	3481.01	1.145	6.583	0.033	5.73	3.16	32.92
Incubation period (96 h)	230	0.037	6084.65	2.395	7.875	0.040	11.98	3.78	39.37
Carbon source (Mannitol)	130	–	–	1.354	–	–	6.77	–	–
Organic nitrogen (Urea)	140	–	–	1.458	–	–	7.29	–	–
Inorganic nitrogen (NH_4_S_2_O_8_)	370	–	–	3.854	–	–	19.27	–	–
Amino acid (Isoleucine)	340	0.098	3469.38	3.541	20.41	0.102	17.71	9.80	102.08
Metal ion (K^+^)	330	0.197	1671.73	3.437	41.12	0.017	17.19	19.74	205.62
Vitamins (Riboflavin, 10 mg)	740	0.396	1868.68	7.708	82.5	0.412	38.54	39.60	412.50
Inoculums size (2 %), SSF	5500	–	–	57.29	–	–	–	–	–
Moisture content (90 %), SSF	13,000	–	–	135.42	–	–	–	–	–

## Conclusion

This study revealed the possibilities of effective utilization of agro-wastes in fermentation (LSSF and SSF) processes as potential substrates where they can act as carbon, nitrogen sources and ultimately produced industrially pertinent enzymes. Obviously, such amalgamation of processes would not only facilitate to trim down the entire production cost, but also focus on the approaches towards effective management of agro-wastes. This study portrays a gainful, expedient, non-tedious, easier technique to scale up for enhanced production of pectinase.
